# New Thinking on the Etiology and Pathogenesis of Late-Onset Alzheimer's Disease

**DOI:** 10.4061/2011/848395

**Published:** 2011-09-18

**Authors:** Alan P. Hudson, Brian J. Balin, Keith Crutcher, Stephen Robinson

**Affiliations:** ^1^Department of Immunology and Microbiology, Wayne State University School of Medicine, Detroit, MI 48201, USA; ^2^Department of Pathology, Immunology, and Microbiology, Philadelphia College of Osteopathic Medicine, Philadelphia, PA 19131, USA; ^3^Division of Science, Aslan Academy, Cincinnati OH 45229, USA; ^4^School of Psychology and Psychiatry, Monash University, Clayton, VIC 3800, Australia

For many years, it has been accepted that the etiology of early-onset Alzheimer's disease (AD) is genetic in nature, with the relevant mutations involving genes encoding products that function in pathways dealing with processing of *β*-amyloid. The etiology of the far more prevalent late-onset AD is not primarily genetic in origin even though research over the last two decades has identified one or more genetic risk factors that predispose their bearers to development of the disease. Studies of late-onset AD have been strongly influenced by the amyloid cascade hypothesis. This influential hypothesis posits that plaques of *β*-amyloid (i.e., neuritic senile plaques, NSP) accumulate in the neuropil, where they eventually initiate a neuropathogenic process that engenders production of insoluble tangles of modified *tau* protein (i.e., neurofibrillar tangles, NFT), the ultimate result of which is progressive cognitive dysfunction. Firm and final diagnosis of the disease remains dependent on *postmortem* quantitation of the density and character of NSP and NFT in specific regions (e.g., hippocampus) of the affected brain. In addition, inflammation in the late-onset AD brain has been well documented, but this potentially important aspect of pathogenesis has not found a major place in the suite of factors thought to play primary roles in the neuropathogenic process. 

Substantial evidence derived from clinical trials, animal model studies, and other sources indicates that late-onset AD cannot be explained solely or primarily by accumulating NSP and NFT despite the fact that these are the most obvious and consistent pathological features of this disease. Rather, the evidence strongly suggests that late-onset AD results from a complex interplay between genetic and environmental factors, most of which remain to be elucidated. In this special issue, we were interested particularly in papers that present alternative viewpoints concerning the complex etiology and pathogenic processes underlying late-onset AD. The solicitation targeted reports of primary research from different fields, including new genome sequence or structure data that shed light on the complex genetic background giving increased susceptibility to disease induction, new insights regarding relevant environmental influences that may contribute to that induction, and new studies focusing on the biochemistry and molecular genetics of AD. Further, we wanted review articles that summarized recent research developments that engender new views on the etiology and/or neuropathogenic mechanisms of AD as well as hypothesis-driven but evidence-based arguments regarding etiology and neuropathogenesis.

The first paper included in this special issue presents an interesting, and we think cogent, argument for a reconsideration of the amyloid cascade hypothesis. The authors of this review conclude that current evidence indicates that the production of NFT and NSP is independent of one another and that these are probably the products, not the cause, of the neurodegeneration that characterizes late-onset AD. The article presents a modified version of the hypothesis that provides a reasonable explanation of the pathogenesis of the disease. 

Since firm diagnosis of late-onset AD depends on *postmortem* examination of the brain of affected individuals, a pressing need exists for identification of markers that can reliably indicate the presence of AD in individuals at much earlier stages of the disease induction process. The second paper included in this special issue presents new research indicating that delineation of certain aspects of the IL-10 genotype and expression of one membrane-located adenosine receptor might prove useful in identifying individuals with the early signs of dementia who are at high risk of progressing to incipient late-onset AD. 

The final three papers accept the amyloid cascade hypothesis as a mechanistic explanation for the characteristic neuropathogenesis of late-onset AD, but each develops an interesting new idea concerning how the accumulation of *β*-amyloid at high levels might be initiated and maintained. The first of the three papers reviews the biochemical roles of the transglutaminase enzymes, the long-standing hypothesis that the activity of these enzymes contributes to oligomerization and accumulation of *β*-amyloid, and posits that specific inhibitors of the enzymes found in the CNS may prove to be effective therapeutic targets to ameliorate disease progression. 

The next paper reports that when A*β*1-42 is infused into mice, the activity of a particular ABC transport system is impaired, leading to attenuated removal of *β*-amyloid from the brain. This intriguing observation suggests that the documented age-related decrease in the expression of that transporter may contribute to the accumulation of *β*-amyloid in the brain and thus of NSP formation. The final paper in this special issue is a study of the function(s) of a noncoding transcript from the BACE1-encoding gene, using a transgenic mouse model in which A*β* production is excessive. Previous work from this group had demonstrated that these noncoding transcripts stabilize the mRNA specifying BACE1, thereby increasing the production of insoluble *β*-amyloid. In the present study, knockdown-based inhibition of either the noncoding or BACE1-encoding transcript engendered decreased *β*-amyloid production and attenuated neuronal degeneration. These last three papers point to new ways of reducing the burden of NSPs in the brains of transgenic mice. The challenge now is to determine if these approaches can be applied to the brains of aging primates, and if so, whether reducing the burden of NSPs will be sufficient to slow the rate of cognitive decline.

AD and stroke together account for the bulk of cognitive impairment and mortality worldwide. As the risk factors and causes of stroke are becoming better understood, interventions have started to decrease the incidence of stroke (see, e.g., [1]). By contrast, despite three decades of intense research, the etiology of late-onset AD remains to be elucidated, and the incidence of this disease is steadily increasing (see, e.g., [2]). The present collection of papers illustrates that progress is being made on several fronts and indicates that many avenues of enquiry remain to be explored. We hope that these papers will stimulate new lines of thinking and experimentation, thereby hastening our understanding of this cruel and insidious disease. 

*Alan P. Hudson* *Alan P. Hudson* *Brian J. Balin* *Brian J. Balin* *Keith Crutcher* *Keith Crutcher* *Stephen Robinson* *Stephen Robinson*
